# Supplementary material for: From sequence to enzyme mechanism using multi-label machine learning
Source: BMC Bioinformatics. 2014 May 19;15:150. doi: 10.1186/1471-2105-15-150 (PMC4229970; doi:10.1186/1471-2105-15-150)
Supplement: Additional file 2 — Java code of ml2db. Additional file ml2db_code.tar.gz contains the Java source code to run the multi-label machine learning experiments and save the results to database. The code’s Javadoc is included. [file 1471-2105-15-150-S2.zip › additional file 2/ml2db/ml2db/doc/main/ml2db/al/utility/neighbours/package-use.html]

Uses of Package main.ml2db.al.utility.neighbours


JavaScript is disabled on your browser.


- Overview
- Package
- Class
- Use
- Tree
- Deprecated
- Index
- Help

- Prev
- Next

- Frames
- No Frames

- All Classes

# Uses of Package main.ml2db.al.utility.neighbours

- Packages that use main.ml2db.al.utility.neighbours

  | Package | Description |
  | --- | --- |
  |  |  |
  | --- | --- |
  | main.ml2db.al.utility.neighbours |  |
  | main.ml2db.al.utility.pickers.byfrequency |  |
  | main.ml2db.graphviz |  |
  | main.ml2db.mulan.mulanarff |  |
  | test.ml2db.al.utility.neighbourhood |  |
- Classes in main.ml2db.al.utility.neighbours used by main.ml2db.al.utility.neighbours

  Class and Description

  LinearMultiLabelNNSearch

  Class

  LinearNNSearchSkipInstanceItself

  Class

  Neighbourhood

  Contains information about: an instance, how much it is needed in this data
  set (the instances it is the nearest neighbour of), and how much it is
  labelled (whether it or its nearest or further neighbours are already
  labelled or partially known).

  NeighbourhoodNetwork

  A collection of instances "needed" to label other instances.
- Classes in main.ml2db.al.utility.neighbours used by main.ml2db.al.utility.pickers.byfrequency

  Class and Description

  LinearNNSearchSkipInstanceItself

  Class
- Classes in main.ml2db.al.utility.neighbours used by main.ml2db.graphviz

  Class and Description

  NeighbourhoodNetwork

  A collection of instances "needed" to label other instances.
- Classes in main.ml2db.al.utility.neighbours used by main.ml2db.mulan.mulanarff

  Class and Description

  LinearNNSearchSkipInstanceItself

  Class
- Classes in main.ml2db.al.utility.neighbours used by test.ml2db.al.utility.neighbourhood

  Class and Description

  NeighbourhoodNetwork

  A collection of instances "needed" to label other instances.

- Overview
- Package
- Class
- Use
- Tree
- Deprecated
- Index
- Help

- Prev
- Next

- Frames
- No Frames

- All Classes
